# Prevalence of Symptoms of Severe Asthma and Allergies in Irish School Children: An ISAAC Protocol Study, 1995–2007

**DOI:** 10.3390/ijerph8083192

**Published:** 2011-08-02

**Authors:** Zubair Kabir, Patrick J. Manning, Jean Holohan, Patrick G. Goodman, Luke Clancy

**Affiliations:** 1 Tobacco Free Research Institute, The Digital Depot, Thomas Street, Dublin 8, Ireland; E-Mails: zkabir@tri.ie (Z.K.); Patrick.goodman@dit.ie (P.G.G.); 2 Royal College of Surgeons in Ireland, 123 St. Stephens Green, Dublin 2, Ireland; E-Mail: pjmanning@eircom.net; 3 Asthma Society of Ireland, 42-43 Amiens Street, Dublin 1, Ireland; E-Mail: Jean.Holohan@asthmasociety.ie; 4 Dublin Institute of Technology, 143-149 Rathmines Road, Dublin 6, Ireland

**Keywords:** allergies, asthma, Ireland, ISAAC, symptoms, severe asthma

## Abstract

Childhood asthma is a recurring health burden and symptoms of severe asthma in children are also emerging as a health and economic issue. This study examined changing patterns in symptoms of severe asthma and allergies (ever eczema and hay fever), using the Irish International Study of Asthma and Allergies in Childhood (ISAAC) protocol. ISAAC is a cross-sectional self-administered questionnaire survey of randomly selected representative post-primary schools. Children aged 13–14 years were studied: 2,670 (in 1995), 2,273 (in 1998), 2,892 (in 2002–2003), and 2,805 (in 2007). Generalized linear modelling using Poisson distribution was employed to compute adjusted prevalence ratios (PR). A 39% significant increase in symptoms of severe asthma was estimated in 2007 relative to the baseline year 1995 (adjusted PR: 1.39 [95% CI: 1.14–1.69]) increasing from 12% in 1995 to 15.3% in 2007. Opposite trends were observed for allergies, showing a decline in 2007, with an initial rise. The potential explanations for such a complex disease pattern whose aetiological hypothesis is still evolving are speculative. Changing environmental factors may be a factor, for instance, an improvement in both outdoor and indoor air quality further reinforcing the hygiene hypothesis but obesity as a disease modifier must also be considered.

## 1. Introduction

Asthma is one of the most common non-communicable diseases in children, and its prevalence varies worldwide [1]. The increase in asthma prevalence in developed countries seen at the end of last century has raised concern for the considerable burden of this disease on society as well as individuals. The International Study of Asthma and Allergies in Childhood (ISAAC), using a simple and inexpensive standardized methodology, has provided valuable data on the prevalence of the symptoms of childhood asthma and allergies for international comparison from countries with different socio-economic backgrounds.

Ireland has the fourth highest prevalence of asthma in the World [2]. Approximately 470,000 people have asthma in Ireland and 6,300 people have severe asthma [3]. The European Community Respiratory Health Survey (ECRHS) reported that Ireland has the highest prevalence of severe asthma attacks (6%) over the past 12 months among patients aged 20–44 years across Europe [4]. Asthma can be a persisting and recurring health problem, thus increasing the likelihood of health, social, and economic costs for these individuals, their families and also on the healthcare system. Ireland has the highest rate of asthma-related hospitalizations in Europe [4]. A recent ISAAC Phase Three study also suggested that the prevalence rates of symptoms of severe asthma can be used as surrogates of both disease burden and health care utilization patterns [5].

Furthermore, the prevalence of symptoms of severe asthma has been shown to correlate more strongly than that of current wheeze alone with national asthma hospitalization and mortality rates particularly in 13–14 year age-groups [6]. While the clinical significance of having only one wheezing episode in a year is questionable, the occurrence of frequent, sleep-disturbing or speech-limiting attacks is widely accepted as clinically important. The latter two symptoms, in particular, are highly specific for bronchial hyperresponsiveness to methacholine [7]. Severe or frequent symptoms are also less likely to go unnoticed by subjects who have no family history of asthma and come from deprived family backgrounds [8]. The ENFUMOSA (European Network for Understanding Mechanisms of Severe Asthma) study was the first comprehensive assessment of severe asthma across Europe to conclude that features of severe asthma are distinct from those described for mild-to-moderate disease [9].

Ireland has reported earlier of an increasing trend in ‘ever’ asthma and wheeze symptoms but it is unclear whether symptoms of severe asthma have changed significantly between the four Irish ISAAC waves (1995, 1998, 2002/2003, 2007). As an objective assessment we looked at changing patterns in the usage of any inhaler (not necessarily corticosteroids) across the four surveys. In addition, the changing patterns in prevalence rates of allergic symptoms (hay fever and eczema) in school-children aged 13–14 years across the four Irish ISAAC waves were determined.

## 2. Methods

### 2.1. Study Population

The study population included school children in Ireland aged 13–14 years. Two previous identical waves were undertaken, the first in 1995 and the second in 1998 (both in Spring). Another two similar waves were undertaken, the third between October 2002 and May 2003, and the fourth was conducted in Spring 2007. The International Study on Asthma and Allergies in Children (ISAAC) study is specifically designed to examine for prevalence of asthma, rhinitis and eczema in children aged 13–14 years. Details on the Irish ISAAC study have been reported elsewhere [10,11].

### 2.2. Sampling Design

In the previous 3 surveys, the basic sampling frame consisted of 624 post primary schools, while for the 2007 survey 731 post-primary schools were used as the sampling frame. More importantly, the first two surveys had identical schools while the latter two surveys had two-third schools of the initial two surveys. A few new schools were included in the last two surveys as those schools initially recruited in the first two surveys were either closed or were not eager to participate in the succeeding surveys. Also, the pupil strength and the number of schools in recent years have grown, thereby, increasing the sampling frame for the last survey in particular.

In all the surveys special disability schools and schools with less than 40 pupils of this age group were excluded because of the impracticalities of administrating the surveys. A multi-stage stratified random sampling technique was employed to require at least 3,000 pupils in consistent with the ISAAC protocol. A random sample of schools was selected from the sampling frame, proportionally stratified by size of school, sex of pupils in school (male, female or mixed) and by administrative Health Areas. The stratification procedure ensured that the schools were fully representative of the population across the specified variables. From each school, classes with the greatest proportion of 13–14 year olds were selected with at least 40 pupils in each class.

The identical survey questionnaire, methods, age groups and schools (*n* = 30) of the 1995 survey were included in the second survey in the Spring of 1998. However, for the 2002/2003 survey, of the potential 36 schools to target a population of 3,080 pupils 4 schools chose not to participate, and therefore 10 new schools were included compared to 1995 and 1998 surveys. For the fourth survey in 2007, 39 schools were initially contacted but 35 schools finally participated in the 2007 survey.

### 2.3. Study Instrument

The questionnaires used in all the four waves were self administered under supervision by the researchers on each occasion. In this study, we estimated the prevalence of asthma symptoms based on the responses to the written questionnaire on (a) wheeze in the past 12 months (current wheeze) and (b) frequent or severe episodes of wheeze in the past 12 months (symptoms of severe asthma). Symptoms of severe asthma are defined as those with current wheeze who, according to the written questionnaire, in the past 12 months, have had >4 attacks of wheeze, or >1 night per week sleep disturbance from wheeze, or wheeze affecting speech. This definition is based on previous ISAAC analyses that showed a combination of these features of more severe wheezing episodes correlated more closely with asthma mortality and hospital admissions than current wheeze alone [5,6]. Allergies (‘ever’ eczema and ‘ever’ hay fever symptoms) were defined from the questions “have you ever had eczema/hay fever?”

### 2.4. Statistical Analyses

Only those aged 13–14 years were included in the final analyses, and therefore the total number of respondents for each of the survey years is not identical to previous published Irish ISAAC studies [11]. A generalized linear model was employed using Proc Genmod of SAS software (version 9.1.1, Cary, NC, USA) for computing adjusted prevalence ratios (PR) for each of the survey years, using the first calendar year 1995 as the reference category. A Poisson distribution with a log link was found appropriate for estimating count data and the goodness-of-fit for models showed no over-dispersion. Prevalence ratios were computed instead of odds ratios as the main outcome of interest, namely, the symptoms of severe asthma was not a common occurrence in the study population (∼15% in prevalence). The log regression coefficients thus computed were exponentiated to get the prevalence ratios. 95% confidence intervals (CI) were computed based on the standard error estimates.

In addition, interactions with calendar year were performed for potential covariates such as sex, smoking status of the study participants (current and secondhand smoke exposure), and school distribution for the health outcomes analysed. Also, interaction terms for calendar year and allergies were performed for the main outcome studied, namely, the symptoms for severe asthma. However, the final models did not include any of the above interaction terms analysed as each of the terms was found not significant statistically. Also, no results by sex distribution were shown as no effect modification was observed for any of the health outcomes analysed across the survey years. Nevertheless, adjusted prevalence ratios accounted for all the potential covariates available to the study, namely, sex, medication usage, clustering effect of schools, pet exposure, smoking status (both current and secondhand smoke exposure), and current wheeze and allergies for the main outcome of interest. Interesting to note that no effect modification of school distribution by calendar years was observed on the health outcomes analysed, thus favouring the representativeness of the schools recruited.

### 2.5. Ethics

Ireland (both LC and PJM) was a partner of the international ISAAC survey committee. Irish ISAAC 1995 had ethical approval from Peamount Hospital Ethics Committee (Dublin), ISAAC 1998 and 2002 got ethical approval from the Federated Hospitals (St. James’s and Tallaght Hospitals) Ethics Committee both in Dublin. The last ISAAC survey in 2007 got ethical approval from the Irish College of General Practitioners’ Ethics Committee.

## 3. Results

A total of 10, 647 participants were studied (Table 1). Table 1 also shows that symptoms of severe asthma increased significantly to 39% in 2007 (adjusted prevalence ratio: 1.39 [95% CI: 1.14–1.69]) relative to 1995 from 12% in 1995 to 15.3% in 2007.

As an objective assessment, any inhaler usage for the same periods have also increased significantly of a similar magnitude and direction, with an estimated increase of 31% in 2007 (adjusted PR: 1.31 [95%CI: 1.11–1.56]). However, the observed significant decline in symptoms of severe asthma in 2002 survey year corresponded well in magnitude and direction to any inhaler usage for the same calendar year but the decline was not significant statistically (adjusted PR: 1.00 [95% CI: 0.84–1.19] *vs.* 0.76 [95% CI: 0.62–0.92]) for inhaler usage and symptoms of severe asthma, respectively.

Table 2 shows that symptoms of ever eczema peaked significantly to 40% in 2002 (adjusted prevalence ratio: 1.40 [95% CI: 1.16–1.69]) relative to 1995 from 9.4% in 1995 to 14.4% in 2002 but showed a marginal decline to 13.2% in 2007 (adjusted prevalence ratio: 1.31 [95% CI: 1.11–1.56]).

Table 3 shows that symptoms of hay fever peaked significantly to 29% in 2002 (adjusted prevalence ratio: 1.29 [95% CI: 1.15–1.45]) relative to 1995 from 25% in 1995 to 31.3% in 2002 but showed a larger statistically non-significant decline almost similar in magnitude to the baseline year of 1995 in 2007, namely, 25.2% (adjusted prevalence ratio: 1.05 [95% CI: 0.93–1.20]).

## 4. Discussion

This study highlights that childhood asthma, especially symptoms of severe asthma, in Ireland is still a public health issue when we estimated a significant increase in prevalence of symptoms of severe asthma in 2007 relative to the baseline year 1995 (an estimated increase of 39%). Further, the estimates are similar to the estimates for any inhaler usage for the corresponding years in both magnitude and direction, thus reinforcing the observed increase in the symptoms of severe asthma through a rather crude objective indicator, namely, any inhaler usage. However, a decline was also observed in between the successive years and the estimated decline was significant statistically only for the calendar year 2002. In contrast, opposite trends were observed for allergies. A significant fall in allergies, especially symptoms of ever hay fever was determined in 2007 almost corresponding to the baseline year prevalence. On the contrary, symptoms of ever eczema continued to rise with a deceleration in the most recent year. Such observations are complex and need further explorations in well-defined longitudinal studies. Nonetheless, the observed increase in symptoms of severe asthma can be either attributed to changing environmental factors or to better recognition and diagnosis of asthma in clinical practice or both.

While much of asthma has a genetic basis, the increasing rise in asthma rates is unlikely to be explained by genetic factors alone and may reflect other issues such as changing but unexplained environment factors [1,5], and also due to an increased awareness of the condition particularly in milder disease [12]. One of the striking environmental changes that occurred in Ireland during these study periods from 1995 onwards is the dramatic improvement in urban air quality [13]. Studies have shown consistently that the introduction of banning bituminous coal initially in Dublin and then gradually expanding all across Ireland had an improvement in health outcomes in the general Irish population [14,15]. The next significant legislation was the introduction of the comprehensive workplace smoke-free policy in Ireland in March 2004. Evidence in Ireland also suggests that the comprehensive workplace smoke-free legislation improved health outcomes both in children and in adults [10,16]. A recent study reported a significant decline in current smoking status of Irish children aged 13–14 years between 1995 and 2007 [10]. The same study also concluded that no significant changes occurred to childhood secondhand exposure levels inside homes, using the Irish ISAAC protocol [10]. Recent evidence in Scotland, however, convincingly demonstrated that comprehensive smoke-free legislation did contribute to an 18.2% yearly decline in childhood asthma hospitalization rates [17].

Other environmental lifestyle factors possibly contributing to the observed increase in symptoms of severe asthma may be the increasing upward trend in childhood obesity [18]. A recent 13-year follow-up study showed a significant relationship between adult onset of asthma incidence and obesity [19]. On the physiological level, adipose tissue might be actively involved in inflammatory processes and there might be a causal link between obesity and chronic inflammatory airway disease [20]. A recent review [21] found a fairly consistent association between weight loss and improved asthma, which is an important epidemiological criterion of causality. A recent meta-analysis also showed a 50% increased risk of asthma in overweight/obese individuals, with a dose-response effect [22]. We also reported that obese adults in Ireland are one and half-times more likely to report physician-diagnosed asthma after adjusting for potential covariates, including smoking status [23]. Unfortunately, the Irish ISAAC survey has no information on obesity. 24% of Irish adults >18 years of age are reported to be obese in a recent nationwide survey [24].

There is epidemiological evidence suggesting that sub-optimal asthma management is a global phenomenon [25], and European nations are also included [26]. The launch and the wide dissemination of the Irish evidence-based Asthma Management Guidelines might have contributed to changes in perception [27]. The guidelines support the early use of inhaled anti-inflammatory corticosteroids, an effective anti-inflammatory asthma therapy in children. Furthermore, there may be less awareness of wheeze being a symptom of asthma, even in those with frequent wheezing [28]. Children with undiagnosed frequent symptoms are also more likely to receive inadequate care for their asthma and may fall into a vicious downward spiral of asthma control [28]. But not all of the paradoxical increase in asthma prevalence could be explained by such a phenomenon, especially the apparent reversal in trend in the symptoms of severe asthma in the most recent survey in 2007.

## 5. Strengths and Limitations of the Study

The main strength of the study is a large cross-sectional survey design employing a uniform internationally validated ISAAC methodology. Identical schools for the first two surveys and recruiting two-thirds of the schools initially sampled for the latter two surveys also addresses the issue of selection bias coupled with a proportional random stratified sampling technique adopted for all the four waves. During analyses, the generalized linear modelling accounted for linearity using a non-linear log link for Poisson distribution and the adjustments for potential covariates influencing any underlying temporal patterns together could have addressed salient weaknesses encountered in the design phase. Nonetheless, cross-sectional study designs have inherent methodological limitations, for instance no causal association. Self-reported paediatric health outcomes may be either over or under reported. Detailed information on socio-economic status or on other unmeasured/unidentified confounders such as parental allergies was not available to all the four Irish ISAAC surveys introducing residual confounding. However, adjustments for clustering effect within schools and the lack of effect modification of schools by calendar years on the health outcomes studied further reinforce minimal or no selection bias in terms of socio-economic status.

## 6. Conclusions

In conclusion, this study indicates that symptoms of severe asthma are emerging as both a clinical and a public health problem in Ireland, thus increasing the likelihood of health, social, and economic costs for these children, their families and also on the national healthcare system. Although the observed patterns are complex and merit further explorations through well-designed longitudinal studies, speculations around some potential environmental changes, for instance, a cleaner air may provide additional clues to the hygiene hypothesis. The current improved status of an outdoor and indoor air quality did contribute to a cleaner environment for Irish children but that may be offset by the rising childhood obesity epidemic with long-term childhood health consequences. In short, the aetiological hypothesis of childhood asthma and more recently the symptoms of severe asthma are continually evolving that merits attention.

## Figures and Tables

**Table 1.** Adjusted Prevalence Ratios (PR) of symptoms of severe asthma and inhaler usage among Irish school children aged 13–14 years, 1995–2007 (*n* = 10,647).

**Year**	*Symptoms of Severe Asthma			**Inhaler Usage**

		**Prevalence Ratios (PR)**		**Prevalence Ratios (PR)**
	
	**Prevalence %**	**Unadjusted [95% CI]**	Adjusted** [95% CI]	Adjusted** [95% CI]
**1995 (*n* = 2,671)**	12.0	1.00 (reference)	1.00 (reference)	1.00 (reference)
**1998 (*n* = 2,273)**	11.4	0.95 (0.80–1.11)	0.90 (0.76–1.07)	1.13 (0.96–1.33)
**2002 (*n* = 2,894)**	8.7	0.72 (0.61–0.85) ^a^	0.76 (0.62–0.92) ^a^	1.00 (0.84–1.19)
**2007 (*n* = 2,809)**	15.3	1.27 (1.10–1.47) ^a^	1.39 (1.14–1.69) ^a^	1.31 (1.11–1.56) ^a^

* Respondents with current wheeze who had 4 or more attacks of wheeze in the last year or had 1 or more nights per week sleep disturbance from wheeze in the last year or had wheeze affecting speech in the last year.

** Adjusted simultaneously for sex; medications; pet exposure; current active smoking status alone; secondhand smoke exposure alone; allergies (hay and eczema symptoms ever) [not for inhaler usage]; clustering effect within schools; wheeze alone [not for inhaler usage].

a Statistically significant.

**Table 2.** Unadjusted and adjusted Prevalence Ratios (PR) of symptoms of ever eczema among Irish school children aged 13–14 years, 1995–2007 (*n* = 10,647).

**Year**	**Symptoms of Ever Eczema**		
	**Prevalence %**	**Prevalence Ratios (PR)**	
		**Unadjusted [95% CI]**	Adjusted** [95% CI]
**1995 (**n **= 2,671)**	9.4	1.00 (reference)	1.00 (reference)
**1998 (*n* = 2,273)**	10.4	1.11 (0.93–1.33)	1.06 (0.89–1.27)
**2002 (*n* = 2,894)**	14.4	1.54 (1.32–1.80) ^a^	1.40 (1.16–1.69) ^a^
**2007 (*n* = 2,809)**	13.2	1.41 (1.20–1.65) ^a^	1.31 (1.07–1.59) ^a^

** Adjusted simultaneously for sex; medications; pet exposure; current active smoking status alone; secondhand smoke exposure alone; hay fever symptoms ever; clustering effect within schools; wheeze past 12months; symptoms of severe asthma.

a Statistically significant.

**Table 3.** Unadjusted and adjusted Prevalence Ratios (PR) of symptoms of ever hay fever among Irish school children aged 13–14 years, 1995–2007 (*n* = 10,647).

**Year**	**Symptoms of Ever Hay Fever**		
	**Prevalence %**	**Prevalence Ratios (PR)**	
		**Unadjusted [95% CI]**	Adjusted** [95% CI]
**1995 (*n* = 2,671)**	25.0	1.00 (reference)	1.00 (reference)
**1998 (*n* = 2,273)**	28.7	1.15 (1.03–1.28) ^a^	1.13 (1.01–1.26) ^a^
**2002 (*n* = 2,894)**	31.3	1.25 (1.13–1.38) ^a^	1.29 (1.15–1.45) ^a^
**2007 (*n* = 2,809)**	25.2	1.01 (0.91–1.12)	1.05 (0.93–1.20)

** Adjusted simultaneously for sex; medications; pet exposure; current active smoking status alone; secondhand smoke exposure alone; eczema symptoms ever; clustering effect within schools; wheeze past 12 months; symptoms of severe asthma.

a Statistically significant.
